# Study on curve optimization and structural safety effect of south roof of solar greenhouse in China

**DOI:** 10.1098/rsos.240352

**Published:** 2024-05-08

**Authors:** Wenbin Shi, Li Wang, Yichao Zhang, Guangning Na, Shaoming Li, Lin Han, Feng Wang, Lei Zhang

**Affiliations:** ^1^ College of Life Engineering, Shenyang Institute of Technology, Fushun, Liaoning, 113122 , People's Republic of China; ^2^ College of Mechanical and Power Engineering, Shenyang University of Chemical Technology, Shenyang, Liaoning, 110142 , People's Republic of China; ^3^ College of Horticulture, Shenyang Agricultural University, Shenyang, 110866 , People's Republic of China

**Keywords:** solar greenhouse, shoulder height, double arc, greenhouse structure optimization

## Abstract

To maximize the use of solar energy and increase the building area of solar greenhouses in China, a light radiation model for solar greenhouses is established. This model integrates previous research results with the solar motion principle, meteorological data and the optical properties of materials. The results indicate that optimizing the structural curve of the south roof of the greenhouse improves both internal land utilization and solar capture. After optimization, the internal land utilization rate of the solar greenhouse increased by 42 m^2^, with a respective 15.2 and 0.78% increase in lighting on the southern roof and ground. The light interception by the back wall of the greenhouse was reduced by 0.67%, while the total light interception increased by 2.22%. The research results identify the optimal shoulder height (0.7 m) and overall height (2 m) for the second-generation solar greenhouse in Liaoshen. The optimal curve functions *Y*_1_ and *Y*_2_ for the south roofs of greenhouses are calculated according to the actual construction requirements. This article verifies the structural safety of the solar greenhouse after renovation and shows that optimizing the shoulder height increases the structural stability and safety of the greenhouse.

## 1. Introduction

The Chinese solar greenhouse (CSG) is a greenhouse that can operate year-round without heating equipment. It relies on the north wall to store heat during the day and on the external insulation to maintain the indoor temperature at night, making it widely used in the cold areas of northern China [1,2]. As an eco-friendly and sustainable energy source, solar energy promotes crop growth. The orientation and planting of the greenhouse depend largely on solar transmittance and sun altitude [3,4]. Solar greenhouses demonstrate excellent performance. Greenhouse transmittance is generally more than 60–80%, and the temperature difference between indoor and outdoor can be maintained at 21–25℃ [5]. Solar radiation plays a pivotal role in maintaining the temperature of solar greenhouses and serves as the source for crop photosynthesis. Therefore, the design of a solar greenhouse must address the lighting of the greenhouse to maximize sunlight penetration into its interior [6].

Winter in China is characterized by a low solar altitude angle of only 21.4° on the winter solstice. Therefore, the design requirement for solar greenhouses is to maximize solar energy utilization in winter [7]. Fernandez-Garcia showed that the optimized overall structure of the solar greenhouse and the implementation of a curved shape of the south roof could enhance the total light quantity and average light transmittance [8,9]. Researchers have studied solar parameters and heating requirements for double plastic-covered greenhouses during the growing season [10,11]. Recent global research on CSG provides an unprecedented opportunity to break the logjam of annual production in high latitudes. While foreign studies may not directly apply to China owing to different climate and geographical conditions, they are still of certain value for reference [12–18]. The orientation of the greenhouse also has a great influence on the light interception inside the greenhouse. Studies have shown that, with Shenyang as an example, the interception of sunlight reaches its maximum when the greenhouse is oriented 5°–6° south to west [19]. Three-dimensional shadow obtained by computer simulation can also determine the influence of the greenhouse’s orientation, length and span on solar interception [20]. For the calculation of scattered radiation in the solar radiation model by computer simulation, two methods to establish the scattering ratio of scattering rate are adopted at home and abroad [21]. According to the empirical model, in cloudless weather, scattered solar radiation is calculated based on a certain proportion of direct solar radiation [22]. These calculations are performed using the Ecotect software, which offers a more accurate analysis of indoor lighting [23]. By comparing the virtual light environment presented by the simulation with the actual light environment, it can be concluded that the simulation results are consistent with the real light environment. Therefore, optical environment simulation can be consistent with the actual optical environment to a certain extent and meet the requirements of display optical measurement [24,25]. Through simulation calculations, it is determined that solar energy captured per square metre of greenhouse land area reaches its maximum when the aspect ratio is 4 [26]. Sethi’s proposed solar radiation availability model has been verified, with the predicted and measured values for different greenhouse structures showing good agreement, indicating the model’s suitability for simulation [27]. The most important aspect of the research on greenhouse energy-saving technology is the greenhouse energy-saving design through the optimal greenhouse shape design to increase overall performance [28]. This article introduces a new solar radiation model developed to numerically analyse the effect of different curvatures of the southern roof on solar interception [29]. Zhang *et al*. built a series of mathematical models to calculate the best way for a solar greenhouse to capture solar radiation and the best architectural orientation for overall solar energy capture [30]. Chen *et al*. built a mathematical model to calculate the best light interception for six different greenhouse structures and established a model for total solar radiation, which was used to select the best greenhouse shape for southern China [31]. Sethi built a greenhouse transport model to simulate the solar beam, scattering and ground reflection and to determine the best greenhouse orientation according to different latitudes to maximize solar energy interception [32]. At present, China’s solar greenhouse vegetable production capacity is only 1/20 of that of developed countries, with relatively low economic efficiency and output per unit area. The average production per hectare is only slightly more than 90 000 hectares, which is about 1/8 of the output of Dutch greenhouse areas; there remains much room for an increase in production and the land area. Previous studies have examined the influence of greenhouse wall structure and span height on the internal environment of the greenhouse, but there has been no systematic research on the front roof structure of greenhouses. Because of the popularity of the second-generation Liaoshen solar greenhouse in China and the persistence of many issues, this article, on the basis of previous studies, optimizes the front roof curve of the second-generation solar greenhouse in Liaoshen.

Based on previous studies, the model takes into account the optico-physical parameters and meteorological data specific to the CSG, which improves the accuracy of the calculation results. Through the integration of quantitative statistics on greenhouse solar interception, the research results can provide scientific and quantitative guidance for CSG construction. The results are of great significance for the reconstruction of solar greenhouses.

## 2. Material and methods

### 2.1. Experimental environmental conditions

The primary objective of solar greenhouse design is to maximize the rational use of sunlight. The illumination conditions in the greenhouse are influenced by many factors, such as the geographical orientation of the construction area, outdoor illumination, greenhouse orientation, building parameters, roof shape and covering material. The test greenhouse in this study was the second-generation Liaoshen-type greenhouse at Shenyang Agricultural University (120°E, 40°N). The dimensions of the greenhouse are as follows: length of 60 m, span of 9 m, ridge height of 3.5 m and a horizontal projection of 1.4 m for the rear roof.

The experimental greenhouse shares identical building parameters and external environmental parameters. The specific orientation of the experimental greenhouse is 7° south by west, with a span of 9 m; polyvinyl chloride membrane is used as the covering material for the roof surface and the gable side, while polyolefin-based film covers the south roof. The new film covering the south roof has a light transmittance of 85%. Given that the solar altitude angle is at its lowest in the northern hemisphere winter solstice and post-winter solstice is the main production stage in the greenhouse in the north, it is crucial for the design of the south roof of the greenhouse to meet the lighting requirements of the wet indoor part during winter. Therefore, this article only focuses on the lighting performance of solar greenhouses on the winter solstice. The specific test date selected is 21 December 2020 (winter solstice).

### 2.2. Experimental design

**Figure 1.**
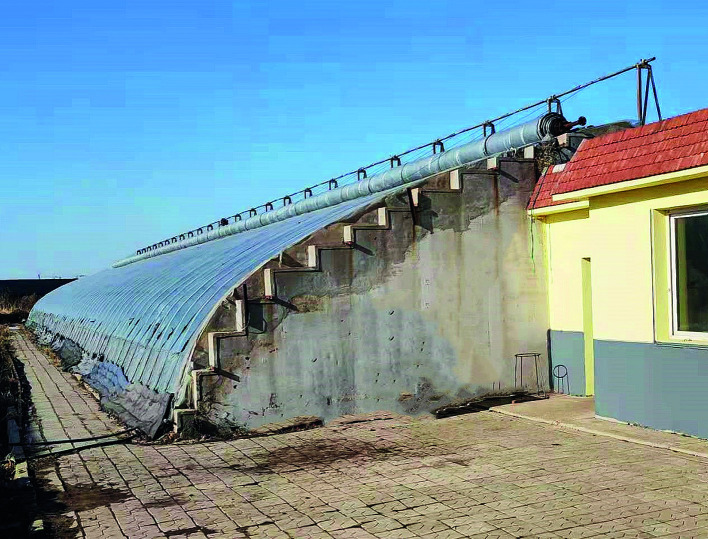
Photo of the solar greenhouse.

The Rhino and Grasshopper software was employed for greenhouse modelling in this experiment, while the Honeybee plugin was used to analyse solar radiation energy within the internal environment of the greenhouse. The obtained results were compared with the actual test outcomes of the greenhouse to validate whether the comparative error fell within an acceptable range. Figure 1 illustrates both the greenhouse and the constructed model for comparison.

The impact of various south roof shapes on the light environment of solar greenhouses was simulated. Previous studies have demonstrated that the cumulative disparity in solar radiation is negligible for roofs with similar shapes [22]. There are four evaluation indexes for the illumination of the greenhouse environment, which include light interception on the south roof of the greenhouse and radiation intensity on each surface inside the greenhouse (interception on the ground, back wall and the greenhouse as a whole).

### 2.3. Theoretical basis

#### 2.3.1. Model building module

The Shenyang area is located at 41.8°N, 12.36°E, characterized by a warm sub-humid continental climate with an annual average temperature of 6.2–9.7℃. When sunlight passes through the films into the solar greenhouse, a portion of the solar radiation is absorbed by the films. The remaining sunlight penetrates the greenhouse interior through membrane refraction, with another fraction directed towards various surfaces within the greenhouse. A portion of this radiation is absorbed by the surrounding air. Each surface within the greenhouse not only undergoes direct absorption of solar radiation and scattered light but also experiences a simulated distribution of radiation in the absence of plant cultivation inside.

**Figure 2.**
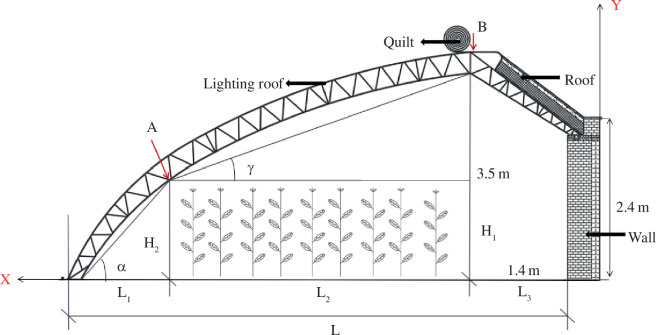
Schematic of measuring points inside the greenhouse and sectional diagram.

Owing to software limitations, the curve of the south roof of the greenhouse was segmented and the circular arc was divided into several continuous lines for modelling. The geometric model was established, as shown in figure 1. Rhino software was used for greenhouse modelling, while the Ladybug and Honeybee software served as a tool for the analysis of greenhouse indoor interior radiation. Therefore, Rhino was selected to design a 1:1 greenhouse simulation model and imported the model into Ladybug and Honeybee software, combined with meteorological data, to obtain hourly radiation values for the greenhouse. To ensure the reliability of the model, the actual optical characteristic parameters of the equipment were set in Honeybee, as shown in table 1. According to figure 2, point A was defined as a new control point: greenhouse shoulder height. Since part of the solar radiation is absorbed by the transparent covering material and another part enters the greenhouse through refraction, the basic parameters used in this model are shown in table 1.

**Table 1.** Parameter list.

basic parameter	parameter values
region	Shenyang
time	2020-12-21
latitude	41°48′11.75″N
longitude	123°25′31.18″E
the length of the greenhouse (m)	60
the width of the greenhouse (m)	9
height (m)	3.5
height of rear wall (m)	2.5
solar constant (w /m^2)^	1367
light transmittance of film	85%
reflective coefficient of rear wall (%)	10
ground reflection coefficient (%)	10

#### 2.3.2. Establishment of the lighting roof curve

Previous studies have shown a mathematical model for the curve of the CSG to select critical control points on the south roof under the same shape of roof lighting, as shown in figure 2. One such point is in the bottom corner in front of the CSG South Room, which corresponds to the critical height for manual labour operation, with point A fixed at (1 m, 1. 3 m). The equation is established as follows:

According to the above lighting roof curve equations, the formula for calculating the total solar energy captured on the lighting roof surface (IT) is given by [27].


(2.1) $$J_{n}=\left(\frac{J_{0}}{r^{2}}\right)P^{csch},$$

(2.2) $$P=\frac{J_{h=90^{\circ }}}{J_{o}},$$

(2.3) $$J_{w,\theta }=j_{n}\left[sinh\;cos\theta +cosh\;sin\theta \;cos\left(A-\partial \right)\right].$$

The solar radiation constant at position $J_{0}$ is 1353 W m^−2^, *r* is the radius of the earth. $J_{w,\theta }$ is the direct radiation intercepted on the covering surface of the greenhouse, $J_{\text{n}}$ is the direct radiation from the normal plane, *h* is the solar altitude angle, *A* is the solar azimuth angle, α is the orientation of the greenhouse and *θ* is the greenhouse roof angle.

#### 2.3.3. Solar radiation intensity in CSG

The solar radiation entering the solar greenhouse is mainly divided into two parts: the solar radiation energy intercepted by the wall and the solar radiation energy intercepted by the ground. The transmissivity of the lighting roof is one of the factors that influences the amount of solar radiation intercepted in CSG. The solar radiation energy intercepted by the wall and ground can be calculated using equations (2.2)–(2.5), respectively [28].


(2.4) $$I_{b,g}=I_{b}g,$$

(2.5) $$I_{b,w}=\frac{cosA}{cosB}I_{b,g},$$

(2.6) $$I_{b,r}=\frac{cosC}{cosB}I_{b,g}.$$

Types *I*_*b*_ *_,g_, I_b,w_* and *I_b,r_* are the direct solar radiation intensity of the soil surface, the surface of the north wall and the rear roof, respectively (W m^−2^), *g* is the transmittance of direct light, A and C are the incidence angle of the solar light on the north wall and the rear roof, respectively.

#### 2.3.4. Data collection and analysis

The experiment was conducted on 21 December 2020, which coincided with the winter solstice. Five representative measurement points were strategically chosen at the central location within the greenhouse (figure 2). The data were measured using a hand-held TES-1339 with a measuring range of 0.01–999, 900 Lux, an accuracy of ±3%, a reading of ±5 bits, and a measuring speed of about five times/s. The data were processed and analysed using Origin and Honeybee.

#### 2.3.5. Simulation results and analysis

**Figure 3.**
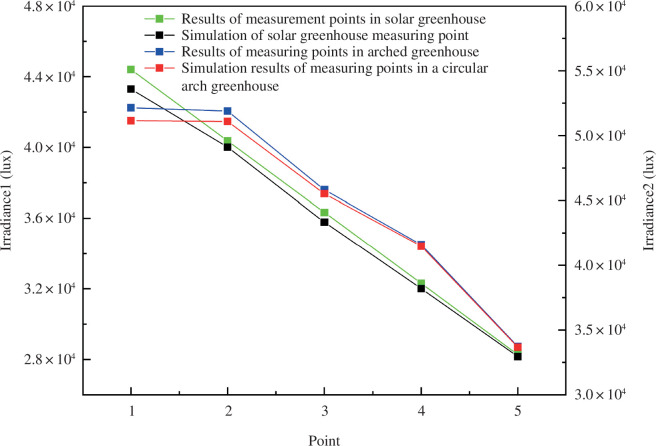
Simulation and measured values of greenhouse ground radiation.

By default, the experiment was set for 12:00 noon on 22 December (winter solstice). Equation (2.4) is used to calculate the predicted value of the CSG subaerial solar intercept captured. The sunlight greenhouse radiation simulation model is established based on various influencing factors, and radiation simulation results were compared and validated using fixed-point test data, as shown in figure 2. To check the reliability of the established model, the simulated values are compared with the measured values from two greenhouses (figure 3). For Model 1, which represents the experiment verification type 2 for arch greenhouse, the correlation coefficient of Model 1 is 0.9, and for Model 2, it is 0.92. These coefficients indicate that the dynamic change of the simulated value is consistent with the measured value, suggesting a good simulation effect. The results show that the model can accurately simulate the radiation distribution in the solar greenhouse to a certain extent.

## 3. Results

### 3.1. Simplified south roof curve model

**Figure 4.**
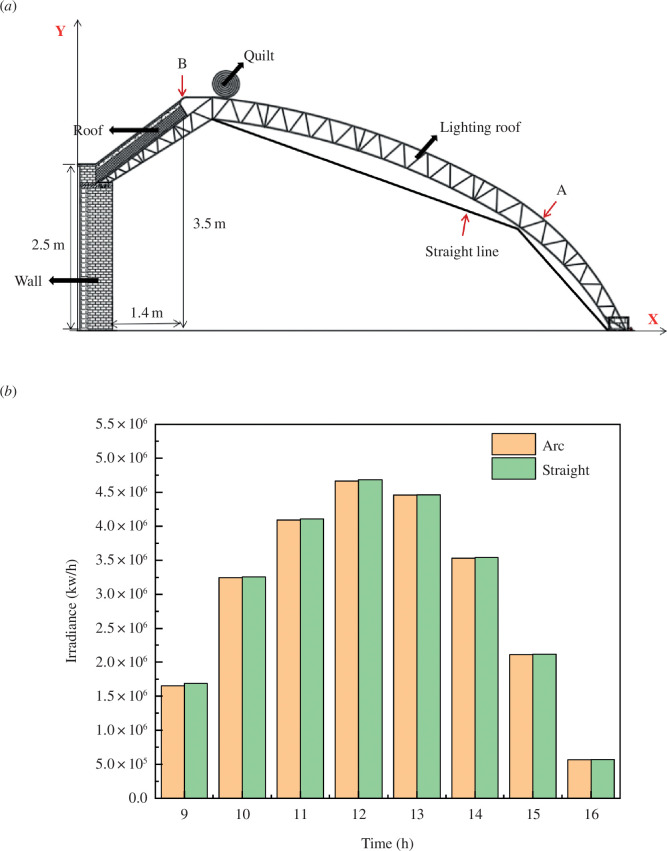
Comparison of two different front roof curves and greenhouse light interception. (*a*) Greenhouse structure diagram; (*b*) front roof structure curve and linear simulation analysis.

By figure 4, it can be inferred that the coordinates of point A (1.4/1.6) simplify the greenhouse roof curve before the greenhouse illumination simulation. Two points from point A to B and C, respectively, set up two straight lines, lines in two linear BA/AC, as shown in figure 4. The simulation is simplified after two straight roofs with circular arcs form different greenhouse before the internal light interception of greenhouse roof conditions. As shown in figure 4*a*, the linear diagram shows the optimization of the south roof of the greenhouse, and figure 4*b* shows the comparison of light interception inside the greenhouse under two conditions.

As shown in figure 4*b*, the optical interception of the optimized two-segment straight line closely aligns with that of the circular arc. Consequently, the optimized straight line will be used for simulations in subsequent tests.

Tomatoes are being cultivated as a case study within the greenhouse. With a tomato plant height of 1.6 m as a reference, it was observed that the area in front of the greenhouse, up to this height (represented as L_1_ = 1.4 m), is too low for crop cultivation. This results in a significant wastage of greenhouse space. To address this issue and enable effective cultivation in the greenhouse, a new south roof curve was designed without altering the height (H_1_ = 1.6 m). The new design involves varying the value of L_1_, which was set to 1.4, 1.15, 0.7 and 0.35 to determine the optimal configuration.

**Figure 5.**
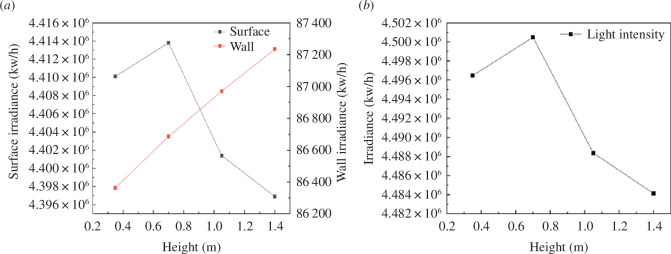
Light interception of greenhouse ground in an hour under different conditions of L_1._

Figure 5 illustrates the simulation results of overall light interception on the greenhouse ground over 1 hour under various L_1_ conditions. As observed in figure 5, the area of the greenhouse ground increases as L_1_ decreases, while the light interception on the back wall decreases in correspondence with that in L_1_. The overall trend of light interception in the greenhouse aligns with that of the ground. When L_1_ is set to 0.7 m, the light interception reaches its peak. This finding indicates that the optimal position for the south roof curve of the greenhouse is achieved when L_1_ is set to 0.7 m.

### 3.2. The effect of the radiative illuminance of the south roof of the greenhouse on the illumination environment of the solar greenhouse

Changes in the shoulder height of the greenhouse become necessary when L_1_ is set to 0.7 m. According to the optimal design theory for the south roof curve, it is imperative to ensure that the minimum angle (γ) remains below 15°, as exceeding this angle would make it difficult to remove the greenhouse insulation covering.

To explore the optimal configuration for the south roof of the double-segment arc solar greenhouse, seven distinct greenhouses with varying shoulder heights were designed (shoulder height positions are indicated in figure 2). These shoulder heights were set to 1.3, 1.4, 1.5, 1.6, 1.7, 1.8, 1.9, and 2.0 m.

**Figure 6.**
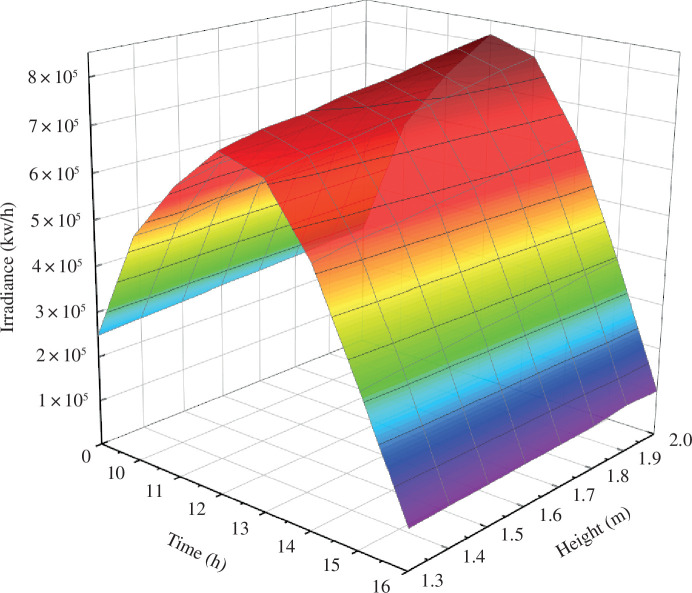
Time-sharing variation law of south roof of greenhouse under different shoulder heights.

Using the model software, the intensity of intercepted light on the greenhouse lighting surface was computed at different times throughout the day (from 09.00 to 16.00 on 21 December) under differing ridge heights, as demonstrated in figure 6.

The illumination levels on each surface within the solar greenhouse serve as vital metrics for indoor lighting intensity. For plant growth, it is essential to meet the minimum requirements of indoor illumination. As evident in figure 6, typical weather conditions result in lower irradiance during the morning and evening hours in winter, making noon a critical period for crop illumination.

An analysis of the lighting effect from 09.00 to 1600 reveals that as the shoulder height increases successively from 1.3 to 2.0 m, the total irradiance on the south roof of the solar greenhouse also increases accordingly.

**Figure 7.**
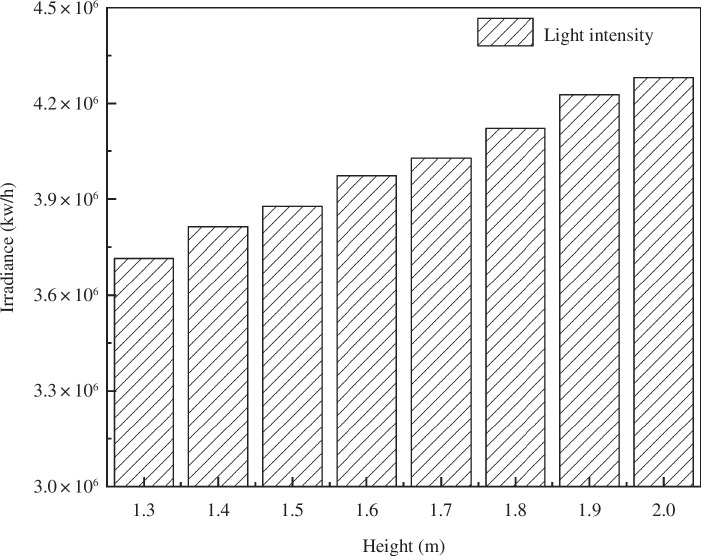
Changes in the total light intensity on the south roof of the greenhouse under different shoulder heights.

Figure 7 demonstrates that the lighting performance of the solar greenhouse improves as the shoulder height increases compared to the control greenhouse. With the incremental rise in shoulder height from 1.3 to 2.0 m, the overall illumination can be enhanced by up to 15.2%. Correspondingly, the indoor irradiance increases by 565,019.59 kW h^−1^, underscoring the conclusion that as the south roof angle continues to be adjusted upwards, the range of light intensity interception by the south roof undergoes an exponential increase.

### 3.3. The effect of the radiative illuminance on the illumination environment of the solar greenhouse

**Figure 8.**
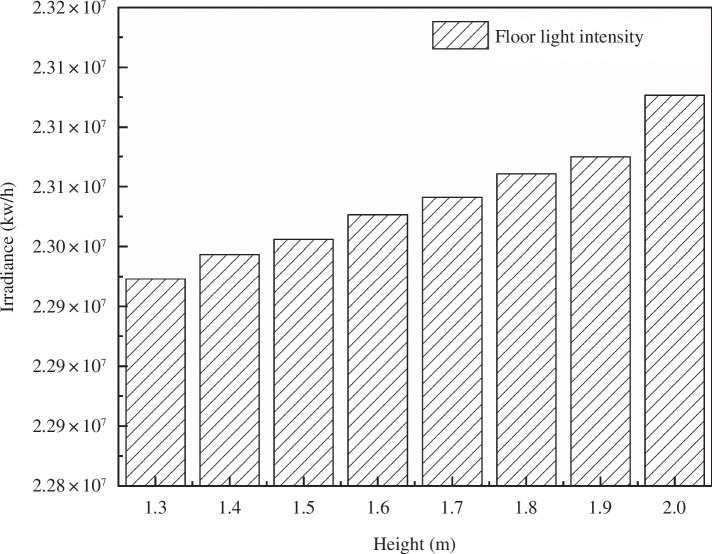
Photonic illumination of greenhouse ground photosynthetically active radiation.

Using the model software, the average photosynthetic photon flux (PAR) illumination on the greenhouse floor was calculated at a specific time (12:00 noon, true solar time on 21 December) under various ridge heights, as presented in figure 8.

Considering the practical conditions of greenhouse production, it can be inferred that solar greenhouses with increased shoulder heights offer superior lighting results compared to control greenhouses. With each incremental increase in shoulder height, the overall lighting rate can be improved by up to 0.78%, with a corresponding increase in indoor irradiance of 180 000 kW h^−1^.

Furthermore, the areas near the east and west sides of the greenhouse are subject to shading from the adjacent walls at different times. Therefore, it is advisable to avoid crop planting directly near the east and west sides of the wall to ensure exposure to sunlight. In the simulation conducted during the test, the solar greenhouse featured a higher shoulder height, resulting in higher indoor irradiance compared to a typical solar greenhouse.

### 3.4. Influence of radiation illumination of the back-wall surface of the greenhouse at a specified time on the illumination environment of the solar greenhouse

**Figure 9.**
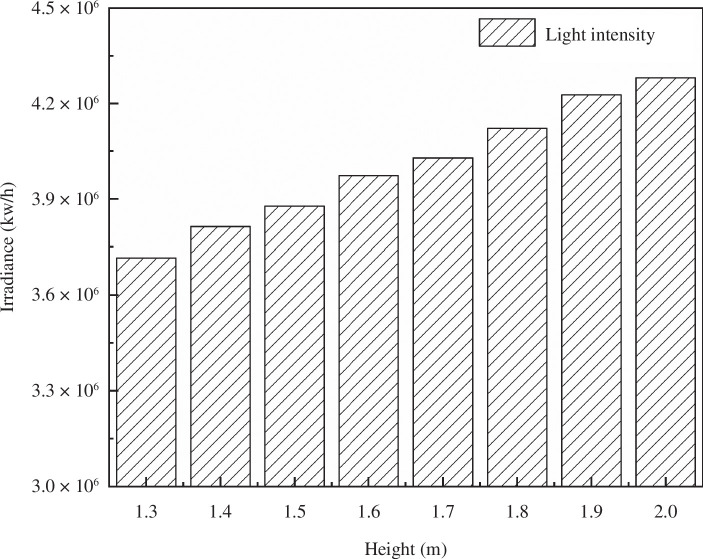
Photonic illumination of photosynthetically active radiation on the back wall of the greenhouse.

According to the model software, the average photonic illumination of PAR on the greenhouse floor at a specified time (12:00 noon, true solar time on 21 December) was calculated under the condition of different ridge heights, as shown in figure 9.

Figure 9 reveals that under typical weather conditions, the analysis of back-wall illumination in the greenhouse from 09.00 to 16.00 shows a decrease in the irradiance on the back wall of the solar greenhouse as the shoulder height increases from 1.3 to 2.0 m. In the indoor solar greenhouse simulated in the experiment, the irradiance of the back wall is lower than that of a standard solar greenhouse owing to the higher shoulder height.

In light of actual greenhouse production conditions, it can be concluded that the lighting performance of the solar greenhouse is enhanced when considering the curvature of the greenhouse lighting surface on the back wall. As the shoulder height increases, the overall lighting rate on the back wall of the greenhouse gradually decreases by 0.67%, along with a corresponding decline in indoor irradiance of 30 000 kW h^−1^.

### 3.5. The effect of the accumulated light radiation energy on the illumination environment of the solar greenhouse

**Figure 10.**
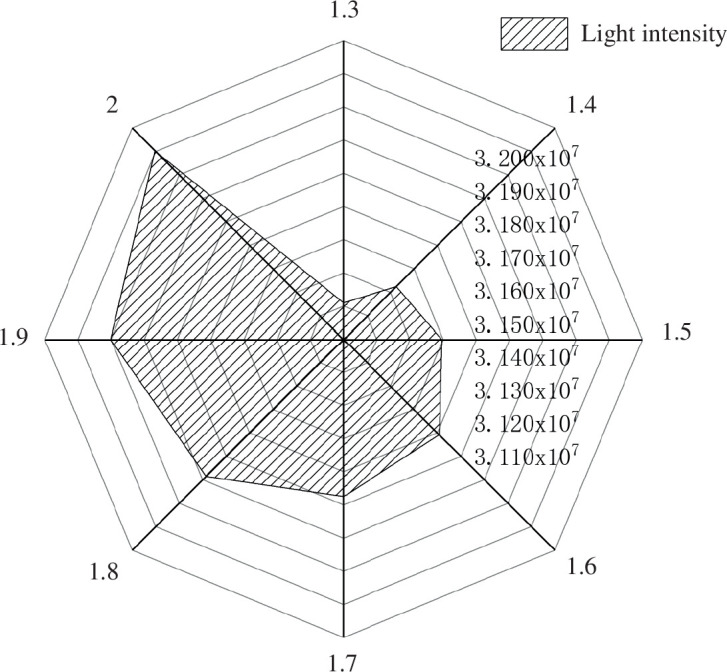
Total amount of greenhouse global effect radiation under different south roof curves.

Using the model software, the PAR on the greenhouse floor and the back wall was calculated at a specific time (12:00 noon on 21 December) under different shoulder heights, as depicted in figure 10.

Figure 10 illustrates the light analysis of the greenhouse floor and the back wall under typical weather conditions. From 09.00 to 16.00, the overall indoor illumination in the solar greenhouse saw a progressive rise, as the shoulder height increased from 1.3 to 2.0 m.

Considering the practical conditions of greenhouse production, it can be inferred that the overall lighting performance of the solar greenhouse, with an increased curvature of the greenhouse lighting surface, surpasses that of the control greenhouse. With the increase in shoulder height, the overall lighting rate within the greenhouse rises by 2.22%, along with a corresponding increase in indoor irradiance to 693 200 kW h^−1^. This observation highlights that when the span of the greenhouse remains constant, a judicious increase in the roof angle is advantageous for a higher total amount of light within the greenhouse.

### 3.6. Variation in light intensity at the ground measurement point under different south roof heights of greenhouse

**Figure 11.**
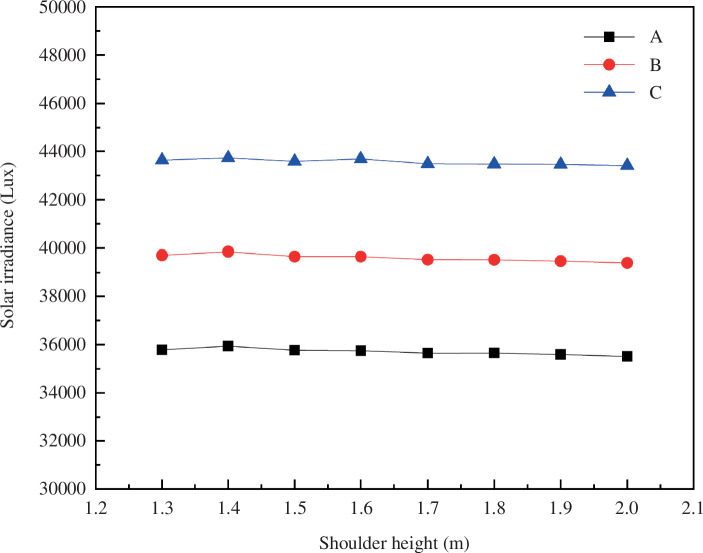
Variation of illumination at the ground point of greenhouse under different shoulder heights.

A higher scattered light ratio is preferable for optimal crop growth within the greenhouse since it ensures more uniform light distribution and a lower risk of localized or uneven growth. As illustrated in figure 11 (A, before the foot; B, middle part; C, after the foot), the ratio of direct to scattered light exhibits a distinct and irregular trend with the increase in roof inclination. This trend signifies that increased roof inclination augments the proportion of scattered light within the greenhouse.

**Figure 12.**
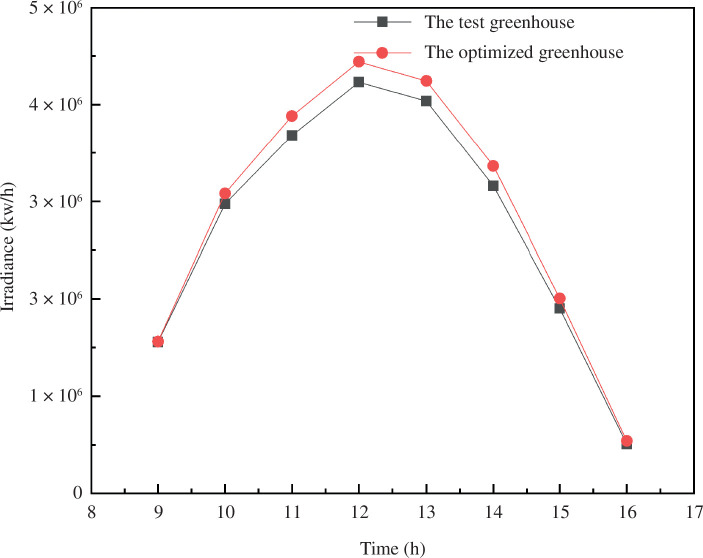
Optimized greenhouse performance comparison.

The experiment identified the optimal shoulder height position for the south roof of the greenhouse to be within the range of (0.7, 2.0). A comparative analysis was conducted on the lighting performance of the successively optimized greenhouses in the south roof area, as illustrated in figure 12. The results showed a significantly improved performance of the optimized south roof greenhouse compared to the unoptimized one. The optimized greenhouse exhibited increased land utilization and lighting performance.

### 3.7. Optimum south roof curve of the greenhouse

**Figure 13.**
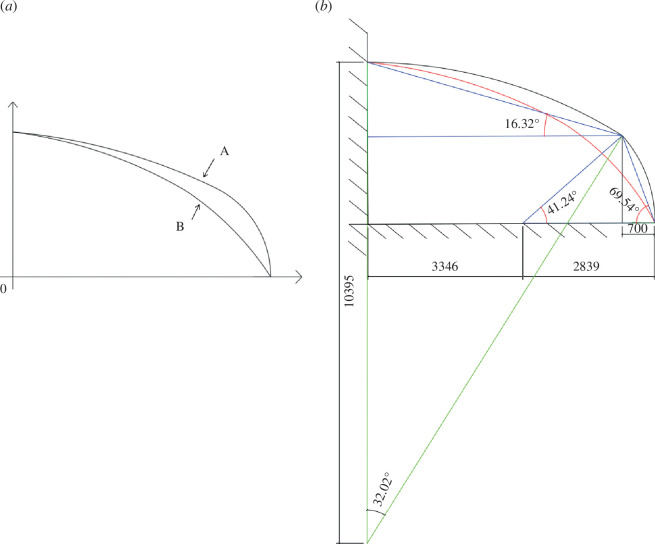
(*a*) Curve (A): post-test curve; curve (B): verification curve. (*b*) Optimize the south roof curve section of the greenhouse.

As depicted in figure 2, L_1_ set to 0.7 m and H_1_ to 2 m results in the optimal lighting roof configuration with γ = 16.48°, α = 75.96°, and a solar altitude angle of 24.84° at noon on the winter solstice in the Shenyang area. During this period, the sun rays are perpendicular to the roof at the front end, without any reflections, which contributes to a superior indoor lighting environment. The optimal position for the shoulder height of the south roof curve in the greenhouse construction is illustrated in figure 13.

Based on the optimal design of the south roof for the greenhouse, the roof is divided into two parts, each fitted with two separate curves. Equation (3.1) determines the upper arc, while equation (3.2) defines the lower one.


(3.1) $$Y_{1}=-0.0271x_{1}^{2}+0.6397x_{1}+3.1808\;R^{2}=0.9996$$

(3.2) $$Y_{2}=-0.1524x_{2}^{2}+3.0362x_{2}+4.9357\;R^{2}=0.9981$$

## 4. Structure safety simulation analysis

The second generation of the solar greenhouse features specific structural parameters, including a 9 m span (L), a 3.5 m ridge height (H), a 1.4 m horizontal projection of the back roof, a 2.4 m back wall height, a 1 m skeleton spacing (B) and a greenhouse roof angle of 38.4°. This greenhouse design incorporates a front arch skeleton with an arched circular roof of approximately 10.3 m in arc length. The rear slope, with an elevation angle of 30.65°, is constructed using a single tube skeleton. Its structure consists of a bottom layer covered by 20 mm thick wood, a middle layer of 150 mm thick benzene board and an outer layer of 4 mm thick waterproof coil. The structure uses 20 mm * 2 mm round tubes for tie rods to connect and secure the components. Transverse tension ribs, made of 20 mm * 2 mm round pipes, are employed with both ends of these ribs linked to the rear slope and front arch frame. Additional parameters are provided in table 2.

**Table 2.** Parameters of material.

materials	ρ/kg m^−3^	elasticity modulus/GPa	Poisson’s ratio	yield strength/MPa
Q235	7850	206	0.3	235

### 4.1. Simulation model building

**Figure 14.**
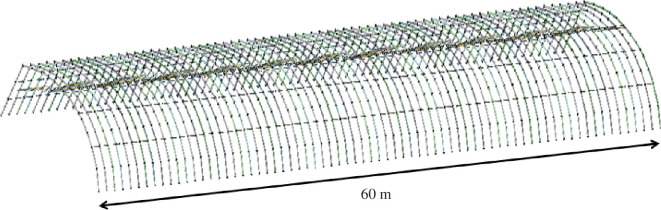
Finite element model.

Beam 188, a three-dimensional two-node beam element incorporating shear-deformation effects based on Timoshenko beam theory, is well suited for this analysis of slender to moderately thick/thick beam structures. As such, it serves as the primary grid division unit for modelling the greenhouse framework. Finite element software is employed for three-dimensional modelling, tailored to the greenhouse’s dimensions. In the modelling process, non-structural factors like the bevelled wall are disregarded to streamline the grid division. Finite element modelling is conducted in alignment with the greenhouse’s structural parameters, as depicted in figure 14.

### 4.2. Mesh generation

**Figure 15.**
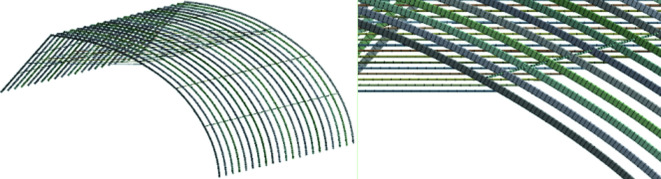
Grid division of greenhouse skeleton.

This article leverages finite element analysis (FEA) software to assess the three-dimensional spatial structure, which provides a comprehensive visualization of stress and deformation distribution under diverse operating conditions. Employing FEA enhances the accuracy and efficiency of structural calculations, offering a precise approximation to the actual deformation, stress and strain experienced by the structure. Therefore, FEA serves as a vital auxiliary tool for greenhouse structure design. The finite element model is created based on the specific structural parameters of the greenhouse, as illustrated in figure 4. The mechanical behaviour of four different types of solar greenhouses using skeletal tubes is analysed through finite element software. Beam188, designed for the analysis of both thin and thick beams and featuring elements based on Timoshenko beam theory with torsional deformation effects, is selected to grid the greenhouse skeleton. In practice, web members mainly endure axial forces. The accuracy and computational time of the analysis are influenced by the density of the mesh in FEA. In this model, each component of the single tube skeleton is divided into one unit, with a grid size of 100 mm for the single tube skeleton in this greenhouse. To enhance calculation precision, the mesh is strategically densified at the joints of structural elements. This article adopts a 100 mm mesh size for the greenhouse grid division, and the impact of the finite element model and mesh size is exemplified in figure 15.

### 4.3. Constraints

The tie rod of the greenhouse skeleton is affixed to the single pipe skeleton through buckles, while the ends of the transverse tension bar are similarly fastened to the front arch and rear slope of the single pipe skeleton via buckles, effectively functioning as rigid constraints. Furthermore, the front roof skeleton and the back roof skeleton of the single tube skeleton structure are welded together to function as rigid constraints. This interconnected greenhouse skeleton structure shares a common node. Additionally, both ends of the skeleton are securely anchored to the rear wall and the front bottom corner foundation. Consequently, the two extremities of the greenhouse framework are designated as fixed points for conducting static mechanical simulation analysis.

Given that greenhouses are typically subjected to various loads, including snow loads and the weight of the rear roof cover, the load distribution on the greenhouse skeleton is intricate. Consequently, this study takes into account the following conditions : (1) constant load + crop load + uniform snow load (a) (table 3).

**Table 3.** The greenhouse skeleton load combination mode.

number	dead load G_k_	snow load S_k_ (kN/m^2^)	wind load (kN/m^2^)	crop load G_k_ (kN/m^2^)	roof live load E_K_ (kN/m^2^)
a	1.0	0.38u_r,b_, 0.38u_r_	--	0.15	0.8

### 4.4. Skeleton-type variable

**Figure 16.**
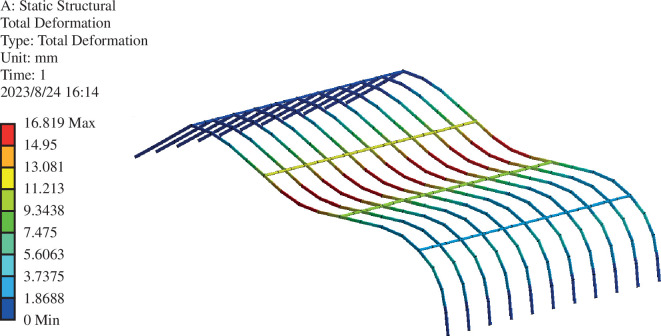
Cloud picture of the roof frame in front of the renovated greenhouse.

**Figure 17.**
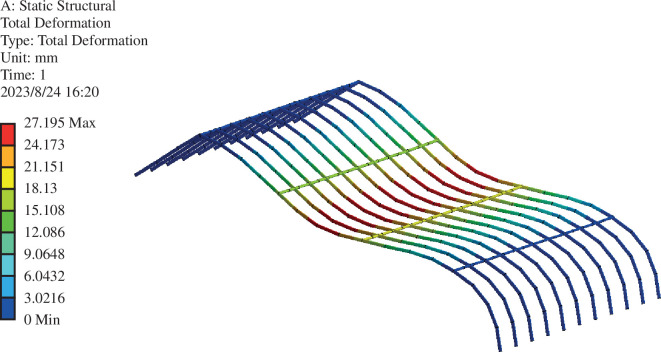
Cloud picture of the front roof frame of the renovated greenhouse.

Based on FEA using ANSYS, contour maps of skeleton-type variables are provided in figures 16 and 17 . As shown in figures 15 and 16, the highest variable in the greenhouse skeleton is located atop the front roof curve, approximately one-third of the way down. The maximum value of the skeleton-type variable in the unmodified greenhouse structure is 27.195 mm, with an average value of 8.7031. After renovation, the maximum value decreases to 16.819 mm, with an average value of 5.1613. These results demonstrate the enhancement of greenhouse structure safety achieved through the optimized front roof curve of the Liaoshen second-generation greenhouse. Additionally, increasing the shoulder height of the greenhouse contributes to safety.

## 5. Conclusion and discussion

This study developed a simulation model of solar greenhouse radiation, which accounted for various influencing factors. The simulation results for ground radiation were compared and validated against experimental observations. The small absolute error indicates that the model can accurately simulate the internal radiation distribution in a solar greenhouse. These findings serve as valuable insights for the establishment of greenhouse microclimate simulation models and the optimization of greenhouse environmental control schemes.

Optimization of the curve height of the south roof in the greenhouse increases the internal land utilization, as it expands the land use area by 42 m² and improves the indoor lighting environment.

Increases in the shoulder height from 1.3 to 2.0 m improve overall illumination by up to 15.2%, along with a corresponding rise in indoor irradiance of 565019.59 kW h^−1^.

Greenhouses with higher shoulder heights exhibit superior ground lighting effects compared to control greenhouses, with a maximum lighting rate increase of 0.78% and a corresponding indoor irradiance increase of 180 000 kW h^−1^.

Higher shoulder heights in solar greenhouses improve lighting on the back wall compared to control greenhouses. Despite a decline in the overall lighting rate of the back wall by 0.67%, the indoor irradiance decreases by 3000 KW/h.

Increases in shoulder height enhance the overall lighting effect of solar greenhouses, along with an increase in lighting rate of 0.46% and a corresponding rise in indoor irradiance of 128239.59 kW h^−1^. This indicates that increasing the south roof’s shoulder height while maintaining the greenhouse span contributes to the total light within the greenhouse.

The study, which verifies the structural safety of the greenhouse’s front roof after renovation, demonstrates that higher shoulder heights enhance the structural stability of the greenhouse and, ultimately, its safety.

In this context, it is advisable to increase the roof shoulder height when keeping parameters like the span of the solar greenhouse fixed. The evaluation index for a solar greenhouse includes the average cumulative irradiance per unit length on various surfaces of the greenhouse during a specific time. Different greenhouse surface spectra, as well as the cumulative total radiation per unit length of the greenhouse, exhibit varying degrees of correlation. Notably, the lighting environment of the ground experiences more significant improvements with increasing roof angle.

Considering the practical aspects of greenhouse production, it can be concluded that raising the shoulder height of the south roof can increase the overall illumination within the greenhouse. Moreover, increasing the shoulder height at the front foot of the greenhouse can expand the land utilization area to significantly increase the planting space for crops and ultimately improve growers’ income.

## Data Availability

Data is available online [33].
